# Proanthocyanidins Delay Fruit Coloring and Softening by Repressing Related Gene Expression during Strawberry (*Fragaria* × *ananassa* Duch.) Ripening

**DOI:** 10.3390/ijms24043139

**Published:** 2023-02-05

**Authors:** Yuanxiu Lin, Chunyan Wang, Shuaipeng Cao, Ziqing Sun, Yunting Zhang, Mengyao Li, Wen He, Yan Wang, Qing Chen, Yong Zhang, Xiaorong Wang, Ya Luo, Haoru Tang

**Affiliations:** 1 College of Horticulture, Sichuan Agricultural University, Chengdu 611130, China; 2 Institute of Pomology & Olericulture, Sichuan Agricultural University, Chengdu 611130, China

**Keywords:** strawberry, proanthocyanidins, softening, ripening, firmness, anthocyanins

## Abstract

Proanthocyanidins (PAs), also known as condensed tannins, are widespread throughout the plant kingdom, presenting diverse biological and biochemical activities. Being one of the most abundant groups of natural polyphenolic antioxidant, PAs are applied to improve plant tolerance to (a)biotic stresses and delay the senescence of fruit by scavenging the reactive oxygen species (ROS) and enhancing antioxidant responses. The effects of PAs on coloring and softening of strawberries (*Fragaria × ananassa* Duch.), a worldwide demanded edible fruit and typical material for studying non-climacteric fruit ripening, were firstly assessed in this work. The results showed that exogenous PAs delayed the decrease in fruit firmness and anthocyanins accumulation but improved the fruit skin brightness. Strawberries treated with PAs had similar total soluble solids, total phenolics, and total flavonoids, but lower titratable acidity content. Moreover, the contents of endogenous PAs, abscisic acid and sucrose, were somehow increased by PA treatment, while no obvious change was found in fructose and glucose content. In addition, the anthocyanin- and firmness-related genes were significantly repressed, while the PA biosynthetic gene (anthocyanin reductase, ANR) was highly up-regulated by PA treatment at the key point for fruit softening and coloring. In summary, the results presented in this study suggest that PAs slow down strawberry coloration and softening by inhibiting the expression of related genes, which could be helpful for a better understanding of the biological role of PAs and provide a new strategy to regulate strawberry ripening.

## 1. Introduction

In addition to being a valuable fruit commodity throughout the world, the strawberry (*Fragaria × ananassa* Duch.) has also been important as a typical model for studies of non-climacteric fruit development and ripening, due to its short vegetative stage and ease of propagation and production. In China, although strawberry fruit can be harvested from November to May of the following year, the fruit harvested in spring (March and April) confers lower economic value traits, including lower firmness, a higher rate of decay, and physiological loss of weight during post-harvest storage, than the fruit harvested in the winter season (November to February) [1]. This is due to the comparatively higher temperature and light intensity in spring, which can further hasten strawberry ripening, and thus, bring poor fruit quality and flavor [2,3]. However, in order to meet consumers’ demand for fruit in different seasons and considering fruit quality, it is very necessary to regulate the ripening of strawberries.

Fruit ripening is an extremely complex process that involves biochemical, physiological and structural changes resulting in fruit coloration, increase in sugar content, decrease in organic acids, formation of flavor and aroma, and fruit softening. This process is developmentally regulated and genetically controlled; the regulatory mechanisms underlying fruit ripening depend on the integrative roles of phytohormones and transcription factors (TFs), as well as epigenetic modifications [4]. In the strawberry, a typical non-climacteric fruit, rather than ethylene and respiration burst, abscisic acid (ABA) and auxin (mainly IAA) are the main hormones that coordinately regulate fruit development and ripening. Over the past decades, the positive regulatory role of ABA on strawberry maturation has been extensively confirmed by various research [5]. On the other hand, it has been demonstrated that the auxin and/or gibberellic acid (GA) inhibit strawberry fruit ripening [6,7]. To date, a large number of TFs belonging to the B-box, bHLH, MYB, MADS-box, and bZIP TF families, have been identified to participate in the strawberry fruit development and ripening process by crosstalk with phytohormones or regulation of related genes [8]. In addition, DNA hypomethylation [9] and N^6^-methyladenosine RNA modification [10] also regulate strawberry fruit ripening. Moreover, some biochemicals or growth regulators have been suggested as positive regulators and widely applied to accelerate strawberry coloration and ripening, such as sucrose [11], melatonin [12], polyamines [13], etc. Additionally, since fruit ripening is an oxidative process that involves alterations in the redox homeostasis of reactive oxygen species (ROS), antioxidants such as ascorbic acid (AsA) and glutathione (GSH) have been suggested to participate in strawberry fruit ripening [14,15,16]. These biochemicals provide multiple ways to regulate strawberry fruit ripening, while the molecular mechanisms still need to be further elucidated.

Proanthocyanidins (PAs), also known as condensed tannins, are the oligomers or polymers of flavan-3-ol units, which have the same typical C6-C3-C6 flavonoid skeletons as anthocyanins. PAs and anthocyanins are both synthesized via the phenylpropanoid and flavonoid pathways; they share common upstream steps and require metabolic intermediates. PAs are mainly made of catechin and epicatechin, while anthocyanins are derived from anthocyanidins by the addition of sugars [17]. The biosynthetic steps have been extensively studied, and it has been suggested that the genes encoding leucoanthocyanidin reductase (LAR) and anthocyanidin reductase (ANR) are key biosynthesis enzymes for PAs, which are regulated by TFs [18]. PAs are natural polyphenolic antioxidants widely found in our dietary foods, especially in fruits and seeds [17,19], and are considered beneficial to human health. It has been extensively suggested that PAs or PA extracts are associated with various bioactivities, exhibiting anti-inflammatory, anti-cancer, and anti-aging effects [20]. Most of these biological effects are mainly due to the high antioxidant capacity of PAs to inhibit hydroperoxide (H_2_O_2_) generation and scavenge ROS, which is about 20 and 50 times higher than that of vitamin C and vitamin E, respectively [21]. In plants, beyond the impacts on fruit quality and taste, usage of PAs can affect various aspects of the plant, such as inhibition of seed germination [22] and improvement of tolerance to biotic and abiotic stresses by regulating the plant antioxidant system to facilitate ROS scavenging [18]. Moreover, the application of exogenous PAs could enhance the biosynthesis of 2-acetyl-1-pyrroline, the key compound of aromatic rice aroma [23]. Recently, Luo et al. [24] have reported that over-production of PAs promoted the ABA formation in roses, indicating the involvement of PAs in ABA signaling modulation. In addition, many studies have indicated that exogenous PA treatment could delay the softening and postharvest ripening of banana fruit [25,26], but the mechanism is far from being clearly explained [27]. However, whether PAs have similar roles in regulating strawberry fruit ripening and related genes expression has not been elucidated so far.

Therefore, in the present study, the effects of PAs on strawberry fruit ripening, as well as the fruit ripening quality, were investigated by exogenous treatment. Furthermore, the expressions of genes involved in the ripening-related anthocyanins biosynthesis and fruit softening were also estimated to explore the potential reason at the molecular level. The results generated in this work will provide a basis for the application of PAs and benefit the regulation of strawberry fruit softening and ripening in a new way.

## 2. Results

### 2.1. Exogenous PA treatment Slows down Strawberry Softening and Coloring

**Figure 1:**
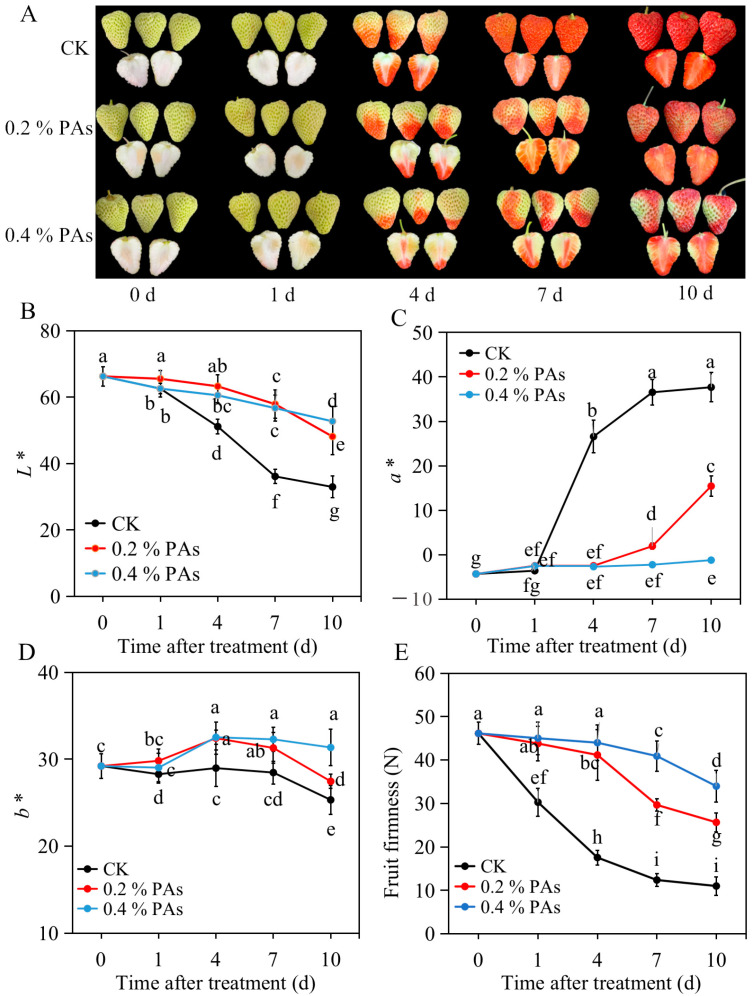
PA treatment affected strawberry ripening. (**A**) PA treatment delayed strawberry coloring. (**B**) Exogenous PAs maintained the L* value of strawberry fruit during postharvest ripening. (**C**) PAs repressed the a* value. (**D**) Change in the b* value under the treatment of PAs. (**E**) PA treatment delayed the decrease in fruit firmness. All data are presented as mean values ± standard deviation. Mean values were obtained from three independent biological replicates. The lowercase letters indicate significant difference based on the LSD multiple test at *p* ≤ 0.05 level.

Fruit colors are usually defined by three parameters in the CIELAB color space: the amount of brightness, denoted by L*; the amount of color from green to red, denoted by a*; and the amount of color from blue to yellow, denoted by b*. As shown in Figure 1A, PA treatment largely delayed the coloration of strawberry fruit, and the 0.4% concentration was more efficient than 0.2% PA. The L* value dropped from 66.3 to 33 on day 10 for control fruit, but from 66.3 to 48 and 53 for 0.2% PA and 0.4% PA treatments, respectively (Figure 1B). This result indicates that PA treatment slowed down the decrease in strawberry brightness. In addition, the PA-treated fruit displayed a significantly lower a* value than that of control fruit (Figure 1C). In control fruit, the a* value sharply increased at 4 d and gradually increased thereafter. On the other hand, a similarly sharp increase was observed in 0.2% PA-treated fruit at 7 d after treatment, while no significant change in a* in 0.4% PA-treated fruit from 1 d to 10 d after treatment was observed. This result suggests at least an approximate 3 d delay in red color formation occurring in the PA-treated fruit compared with the control fruit. Furthermore, the b* value showed a similar trend in control fruit and 0.2% PA-treated fruit, which first exhibited an increase from 0 d to 4 d and then decreased from 4 d to 10 d after treatment (Figure 1D). However, in 0.4% PA-treated fruit, the b* value displayed an overall increase trend from 0 d to 10 d after treatment. Despite this, the b* value in PA-treated fruit was higher than that of the control, and the 0.4% PA-treated fruit showed the highest b* value. Moreover, the firmness of control fruit rapidly decreased 1 d after treatment and kept decreasing to 11 N until 10 d after treatment (Figure 1E), while the firmness in 0.2% or 0.4% PA-treated fruit dropped moderately from 46 to 26 or 34 N at 10 d. The firmness decline in PA-treated fruit was also delayed by about 3 d compared to that in the control fruit.

### 2.2. Total Soluble Solids, Titratable Acidity, Total Phenolic Content, and Total Flavonoid Content Determination

Generally, throughout ripening, the vast majority of fleshy fruits are characterized by increase in sugar contents, whereas titratable acidity (TA) decreases. In our results, overall, total soluble solid (TSS) content showed a gradual decreasing trend, while TA content exhibited a decreasing trend, in both control and PA-treated fruit (Table 1). Specifically, 1 d after treatment, the control fruit showed the highest TSS content, followed by 0.4% PA and 0.2% PA treatments; at 4 d and 7 d, 0.2% PA- and 0.4% PA-treated fruit contained the highest and lowest TSS contents, respectively; while 10 d after treatment, the highest TSS level was found in 0.4% PA-treated fruit, and no distinct change was observed in 0.2% PA-treated and control fruit. In strawberries, it has been suggested that TA increases from 10 days after bloom to the turning stage, or from 21 to 28 days from fruit set, and then decreases until maturity [28,29]. Similarly, in the present study, we found that TA increased from 0 to 4 days after treatment and then decreased until 10 days after treatment. The PA treatment somehow decreased the TA content starting at 4 days after treatment. The TSS/TA ratio dropped from 17 to 6.2 in control fruit, while dropping from 17 to 7.5 or 7.8 in 0.2% PA and 0.4% PA-treated fruit (Table 1). The total flavonoid content (TFC) and total phenolic content (TPC) of the fruit in each comparison group showed a sharp reduction from 0 d to 1 d and then underwent a gradual increase from 1 d to 10 d after treatment. Eventually, the TFC and TPC reached a similar level and displayed no obvious change in each treatment (Table 1).

### 2.3. In Vivo PA and Anthocyanin Content

**Figure 2:**
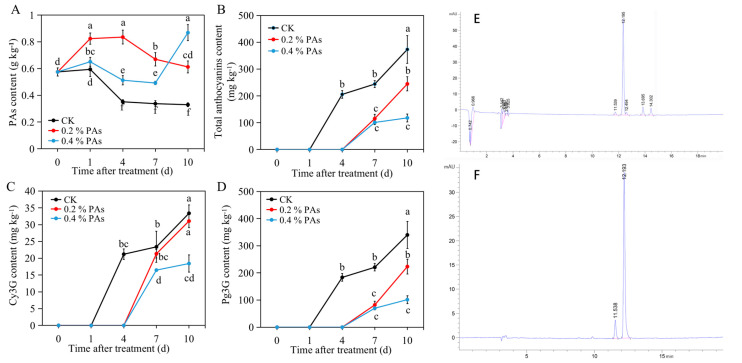
PA content and anthocyanin content in PA-treated and untreated fruit. (**A**) Change in PA content after the exogenous treatment. (**B**) Total anthocyanin content in the PA-treated and untreated fruit. (**C**,**D**) Content of the main anthocyanin components, Cy3G (cyanidin 3-glucoside) and Pg3G (pelargonidin 3-glucoside), in the PA-treated fruit compared to the control. (**E**) HPLC peaks of 0.2% PA-treated sample at 7 d. (**F**) HPLC peaks of anthocyanins standards. The peaks with retention time of around 11.5 and 12.2 min indicate the Cy3G and Pg3G, respectively. All data are presented as mean value ± standard deviation. Mean values were obtained from three independent biological replicates. The lowercase letters indicate a significant difference based on LSD multiple test at *p* ≤ 0.05 level.

PA content in control fruit decreased after treatment, whereas exogenous PA treatment effectively delayed the decline in PA content (Figure 2A). In the 0.2% PA-treated fruit, PA content first increased from 0 d and peaked at 4 d, and then decreased to a level around two times higher than that in control fruit. However, in the 0.4% PA-treated fruit, PA content slightly increased from 0 d to 1 d after treatment, then decreased until reaching 7 d. Eventually, the PA content sharply increased and peaked at 10 d, which exhibited a level about three times higher than that in control fruit.

The two main anthocyanins, cyanidin 3-glucoside (Cy3G) and pelargonidin 3-glucoside (Pg3G), were measured by the HPLC method. It was suggested that anthocyanins started to accumulate at 4 d or 7 d after detachment in the control or PA-treated fruit (Figure 2B), indicating that exogenous PAs delayed the coloration of strawberry fruit. At 10 d after treatment, the control fruit accumulated the highest level of anthocyanins, followed by 0.2% and 0.4% PA-treated fruit. Furthermore, as shown in Figure 2C, the Cy3G content was distinctly higher in control fruit than that in 0.4% PA-treated fruit, while it showed no significant difference between the control and 0.2% PA-treated fruit. For the Pg3G content (Figure 2D), the fruit in the control group contained the highest level, followed by 0.2% and 0.4% PA treatment, except at 7 d, at which no significant difference was found between 0.2% and 0.4% PA-treated fruit. A representation of the HPLC peaks is shown in Figure 2E,F.

### 2.4. PA treatment Altered ABA and Sugar Content

**Figure 3:**
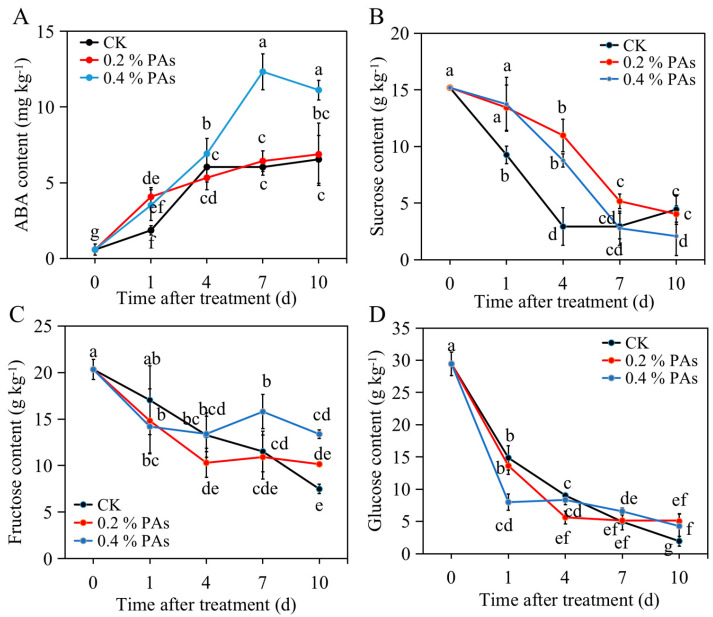
Effects of PA treatment on (**A**) ABA content, (**B**) sucrose, (**C**) fructose, (**D**) glucose content. All data are presented as mean values ± standard deviation. Mean values were obtained from three independent biological replicates. The lowercase letters indicate significant difference based on LSD multiple test at *p* ≤ 0.05 level.

As shown in Figure 3A, the ABA content exhibited a general upward trend in both control and PA-treated fruit. Specifically, it sharply increased and reached a peak at 4 d, and then slightly decreased in control fruit. Meanwhile, a quick increase in ABA content from 0 d to 1 d after PA treatment was found. Under 0.4% PA treatment, ABA content kept increasing from 0 d and peaked at 7 d, followed by a slight decrease from 7 d to 10 d after treatment. In the 0.2% PA-treated fruit, ABA content gradually increased from 0 d to 10 d. The control and 0.2% PA-treated fruit had similar ABA level, while 0.4% PA-treated fruit had higher ABA content from 4 d to 10 d (Figure 3A).

By contrast, the soluble sugars, including sucrose, fructose, and glucose, presented similar downward trends after PA treatment. In particular, the sucrose content in control fruit was lower than that in 0.2% PA- and 0.4% PA-treated fruit from 0 d to 4 d (Figure 3B), which could be possibly a result of the potential repressing effect on the sucrose synthesis genes, such as sucrose synthase or sucrose phosphate synthase, at 10 d after treatment. However, no distinctive accumulation of sucrose at 7 d and 10 d in control and 0.2% PA-treated fruit was found. Eventually, 0.4% PA-treated fruit showed a lower sucrose level than control and 0.2% PA-treated fruit. In addition, the fructose showed a similar decrease trend in control and PA-treated fruit from 0 d to 4 d, while a different trend was observed afterwards (Figure 3C). A continuous decrease and slight increase from 4 d to 10 d after treatment was observed in control and PA-treated fruit respectively. At 10 d, 0.4% PA-treated fruit accumulated the highest fructose level, followed by 0.2% PA-treated and control fruit. Similarly, the glucose content constantly decreased from 0 d to 10 d in control fruit (Figure 3D). However, it rapidly decreased from 0 d to 1 d or 4 d in 0.4% or 0.2% PA-treated fruit, and thereafter slowly decreased in 0.4% PA-treated fruit but maintained at a relatively stable level in 0.2% PA-treated fruit. It was significantly higher in PA-treated fruit than in control fruit at 10 d after treatment (Figure 3D).

### 2.5. PAs Inhibited Anthocyanin-Related Gene Expression at the Key Point for Fruit Coloring

**Figure 4:**
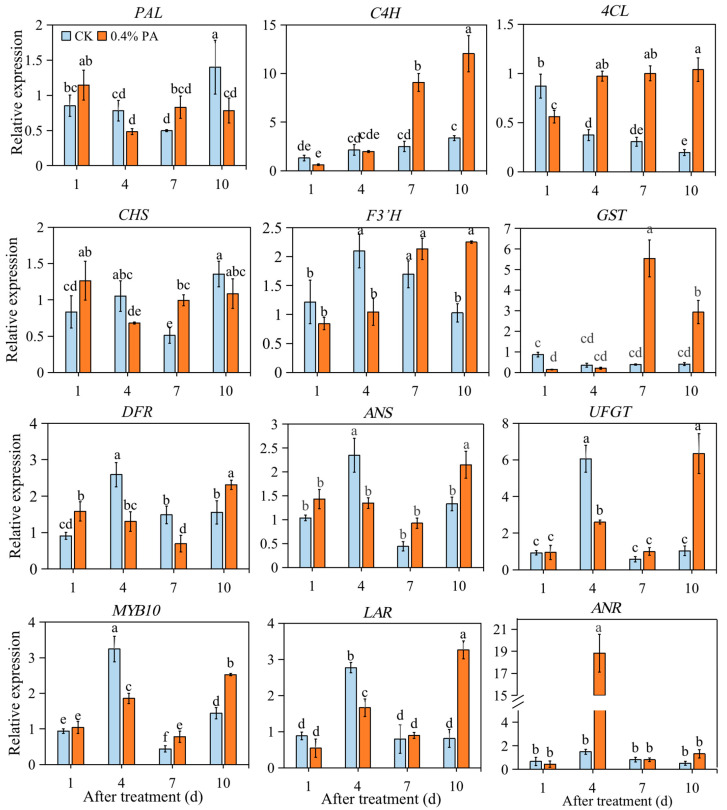
Relative expression of anthocyanins- and PA-related genes under 0.4% PA treatment. *PAL*: phenylalanine ammonia lyase; *C4H*: cinnamate 4-hydroxylase; *4CL*: 4-coumarate:coenzyme A ligase; *CHS*: chalcone synthase; *F3′H*: flavonoid 3′-hydroxylase; *GST*: glutathione transferase; *DFR*: dihydroflavono-4-reductase; *ANS*: anthocyanidin synthase; *UFGT*: UDP-glucose flavonoid-3-O-glycosyltransferase; *LAR*: leucoanthocyanidindin reductase; *ANR*: anthocyanidin reductase. The data are presented as mean values ± standard deviation. Mean values were obtained from three independent biological replicates. The lowercase letters indicate significant difference based on LSD multiple test at *p* ≤ 0.05 level.

Exogenous PAs altered the expression patterns of anthocyanin- and PA-related genes (Figure 4). Compared with the control, the transcript levels of the evaluated anthocyanin-related genes, including phenylalanine ammonia lyase (*PAL*), chalcone synthase (*CHS*), flavonoid 3′-hydroxylase (*F3*′*H*), dihydroflavono-4-reductase (*DFR*), anthocyanidin synthase (*ANS*), UDP-glucose flavonoid-3-O-glycosyltransferase (*UFGT*), and *MYB10,* were repressed at 4 d after the 0.4% PA treatment, upon which the anthocyanins started to accumulate (Figure 1). Thereafter, the relative expression of *F3*′*H*, *ANS*, *UFGT,* and *MYB10* increased to a higher level at 7 d and 10 d; transcript abundances of *PAL* and *CHS* reached to a higher level at 7 d and then decreased to a lower level again at 10 d; the *DFR* expression rose to a higher level until 10 d after treatment comparing to the control. The expression of cinnamate 4-hydroxylase (*C4H*) and glutathione transferase (*GST*) did not show obvious change at 4 d after treatment but exhibited significantly higher levels at 7 and 10 d after treatment, which might be related to the lignin biosynthesis and PA transportation. Notably, the expression of 4-coumarate:coenzyme A ligase (*4CL*) gradually decreased in the control fruit during ripening, while the opposite trend was observed in the PA-treated fruits. Thus, *4CL* showed a significantly higher expression level in PA-treated fruits at 4, 7, and 10 d. Moreover, the expression of *LAR* was also down-regulated at 4 d and significantly up-regulated at 10 d in the PA-treated fruit compared to that in the control fruit. The expression of *LAR* showed a similar level at 1 d and 7 d in PA and control fruit. However, the *ANR* expression was remarkably increased by 0.4% PA treatment at 4 d, while it was similar at 1 d, 7 d, and 10 d after treatment (Figure 4).

### 2.6. PAs Affected the Expression of Firmness-Related Genes

**Figure 5:**
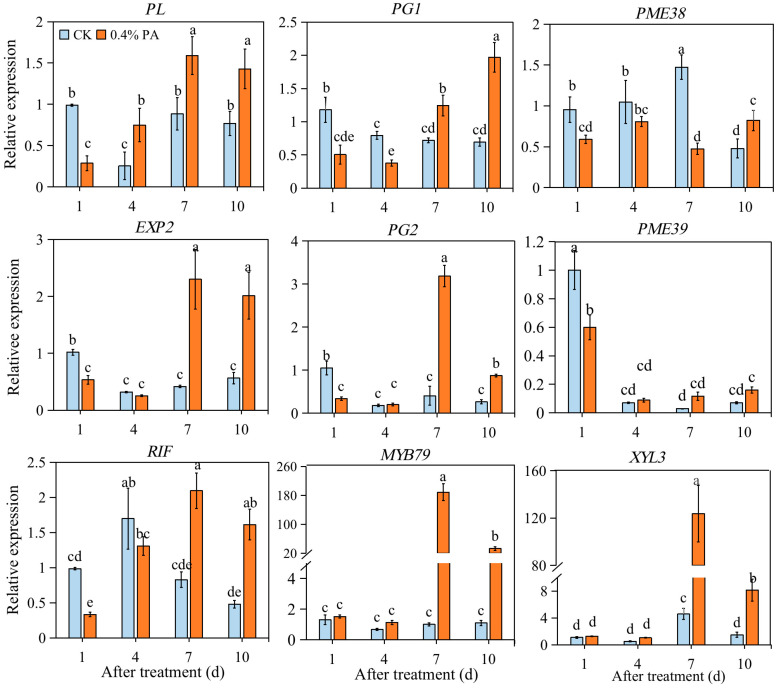
Relative transcript levels of cell-wall-related gene expression under 0.4% PA treatment. *PL*: pectate lyases; *PG*: polygalacturonase; *PME*: pectin methylesterase; *RIF*: ripening inducing factor; *EXP2*: EXPANSIN2; *XYL3*: β-xylosidase. The data are presented as mean values ± standard deviation; the lowercase letters above the bars indicate significant difference at *p* ≤ 0.05 level.

In the control fruit, the expression of the pectate lyases (*PL*) gene sharply decreased at 4 d, and then increased at 7 d and 10 d to a similar level to 1 d. In contrast, it showed a gradual increase trend of *PL* expression under 0.4% PA treatment. Except for 1 d, *PL* gene expression was increased by PA treatment at 4, 7 and 10 d (Figure 5). Polygalacturonases (*PG1* and *PG2*) displayed similar decrease trends in the control fruit, while opposite trends were observed in the 0.4% PA-treated fruit from 1 d to 10 d (Figure 5). The relative expression levels of *PG1* and *PG2* were lower at 1 d and 4 d but higher at 7 d and 10 d in the PA-treated fruit compared to the control. The expression of pectin methylesterase (*PME38*) exhibited a lower level under PA treatment from 1 d to 7 d, while exhibiting a higher level at 10 d. For the expression of *PME39*, it was inhibited at 1 d after PA treatment, while, thereafter, although no statistical changes were observed, it showed a higher level in PA-treatment fruit at 4, 7 and 10 d compared to that in the control fruit (Figure 5).

**Figure 6:**
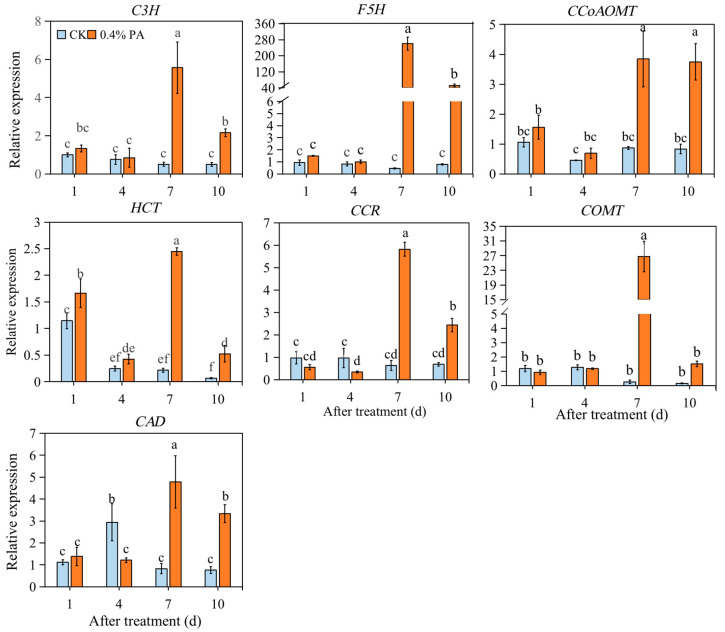
Relative transcript levels of lignin biosynthetic genes under 0.4% PA treatment. *C3H*: coumarate 3-hydroxylase; *F5H*: ferulate 5-hydroxylase; *CCoAOMT*: caffeoyl CoA O-methyl transferase; *HCT*: shikimate O-hydroxycinnamoyl transferase; *CAD*: cinnamyl alcohol dehydrogenase; *CCR*: cinnamoyl CoA reductase; *COMT*: caffeic acid O-methyltransferase. The data are presented as mean values ± standard deviation; the lowercase letters above the bars indicate significant difference at *p* ≤ 0.05 level.

Furthermore, cinnamyl alcohol dehydrogenase (*CAD*) and cinnamoyl CoA reductase (*CCR*) expression showed no significant change at 1 d, but significant repression at 4 d and induction at 7 d and 10 d, respectively in the PA-treated fruit. The caffeic acid O-methyltransferase (*COMT*) expression showed remarkable up-regulation at 7 d after PA treatment, while it showed no significant change at 1 d, 4 d and 10 d in the 0.4% PA-treated fruit (Figure 6). Likely, no evident change in the expression of coumarate 3-hydroxylase (*F5H*), caffeoyl CoA O-methyl transferase (*CCoAOMT*), and coumarate 3-hydroxylase (*C3H*) at 1 and 4 d after PA treatment was found. However, noticeable up-regulation of the expression of *F5H*, *C3H* and *CCoAOMT* occurred at 7 and 10 d after PA treatment. For the expression of *HCT*, a significantly higher level was observed at 1, 7 and 10 d after PA treatment, while a similar level was found at 4 d after PA treatment (Figure 6).

## 3. Discussion

The strawberry is a highly demanded edible fruit, cultivated and consumed worldwide. It is highly favored for its nutritional and medicinal properties. However, strawberry fruits develop and ripen very rapidly, especially in high-temperature environments. It is well known that when the temperature is high, strawberry ripening and coloring occur very quickly, and thus produce smaller fruit with lower quality [30]. Slowing down ripening during periods of heat is necessary, which will benefit the strawberry’s fruit quality. Nowadays, a series of methods have been developed to delay the ripening of strawberries, such as the application of phytohormones [6,7], 5-azacytidine [31], nitric oxide [32], and antioxidants [14,15,16]. In this study, we found that exogenous application of PAs significantly slowed down the softening and coloring of strawberries (Figure 1). Our results here offer a potentially efficient and safe method to delay strawberry ripening in strawberry production. PAs are the second-most abundant polyphenols after anthocyanins in strawberry fruit [33]. They are not only considered beneficial to human health, but also may act both as antifungal compounds and antioxidants to extend fruit shelf life and enhance fruit quality preservation [18,34]. In this study, exogenous PA infiltration delayed the ripening process of strawberries by slowing down the softening and coloring (Figure 1), suggesting a potential new role of PAs involved in fruit ripening.

Strawberry fruit ripening is a complex, genetically and environmentally regulated process. ABA and sucrose were suggested as the main regulators of strawberry fruit ripening. Exogenous ABA and sucrose application largely accelerate strawberry ripening [11]. To estimate whether exogenous PAs delay strawberry ripening by participating in ABA or sucrose signaling, the ABA and sucrose content was detected. In our results, 0.2% PA treatment did not change, while 0.4% PA up-regulated, the ABA content significantly (Figure 3A), making it clear that PAs in certain concentrations could promote ABA production. This is supported by previous studies, which have claimed that PAs promoted ABA biogenesis in *Arabidopsis thaliana* [22]. Meanwhile, our results show that PA treatment increases sucrose content (Figure 3B), which gave us a clue that PAs slow down strawberry fruit ripening, but not by suppressing the ABA and sucrose production.

A series of biochemical and physiological changes takes place during the ripening process. First of all, the degree of fruit coloring is an important index of fruit ripening, and usually expressed in numerical terms along the L*, a*, and b* axes (from white to black, green to red, and blue to yellow, respectively). According to our results (Figure 1), the L*, a*, and b* values were significantly altered by PA treatment, for which, L* and b* values were higher, while the a* value was lower in PA-treated fruit than that in the control fruit during the ripening process. This result indicates a higher brightness but lower redness, and again clearly suggests a delay in fruit coloration of PA-treated fruit. Consistently with this, our results further exhibited an obvious repression of total anthocyanin content in both 0.2% and 0.4% PA-treated fruit (Figure 2B), because the red color of strawberry fruit is the result of anthocyanin accumulation. In addition, the Pg3G and Cy3G are commonly regarded as two major anthocyanins in strawberries; the Pg3G occurs in a higher amounts than Cy3G and accounts for around 70% of total anthocyanins [35]. The HPLC results in the present study also showed that under 0.2% PA treatment, only Pg3G was significantly changed, while the Cy3G showed no change (Figure 2C,D), suggesting 0.2% PAs repressed the fruit coloration mainly by suppressing Pg3G biosynthesis. At the molecular level, anthocyanin accumulation is regulated by the expression of biosynthetic genes and the major regulator *MYB10* [36]. Most of these genes are barely expressed in the early developmental stage and then sharply increase at the turning stage, upon which the anthocyanins start to accumulate [37]. Similarly, in our results (Figure 4), most of the anthocyanin related genes, such as *F3′H*, *DFR*, *ANS*, *UFGT* and *MYB10*, largely increased form 1 d to 4 d in the control fruit, resulting in color formation at 4 d. In contrast, in the PA-treated fruit, the increase in gene expression, including *F3′H*, *DFR* and *ANS,* was delayed to 7 d or 10 d after treatment. Although the expression of *UFGT* and *MYB10* significantly increased at 4 d compared to 1 d, both of the highest levels were detected at 10 d after PA treatment. Structural genes play an important role in flavonoid and anthocyanin biosynthesis. For examples, the antisense repression of *CHS* could lead to colorless strawberry fruit [38]; *FaF3′H* rarely expressed during strawberry fruit development period, and the decreased *FaF3′H* gene expression blocks Cy3G accumulation in red-flesh strawberries [39]; and the lower expression of *ANS* gene has been suggested to contribute to the white color of Chilean strawberries [40]. Therefore, the repression of *ANS*, *F3′H*, *DFR,* and *CHS* expression at 4 d after PA treatment, but a subsequent increase in these genes’ expression at 7 d after treatment, might provide one of the possible reasons that PA treatment delayed anthocyanin accumulation from 4 to 7 d. Otherwise, the upstream gene *4CL* was largely induced from 4 to 10 d after PA treatment (Figure 4), which might contribute to the precursors for the downstream lignin biosynthesis pathway and fruit firmness, because anthocyanin and lignin biosynthesis share the same upstream phenylpropanoid pathway.

On the other hand, the apparently higher PA content might contribute to the low anthocyanins in the PA-treated fruit as well (Figure 2A). Since the PAs and anthocyanins were synthesized by competitive branches derived from the common phenylalanine pathway, it was previously suggested that overexpression of the regulators of PA biosynthesis in strawberries resulted in a loss of red coloration in the flesh, accompanied with an increased PA content [41], while the silencing of the glucosyltransferase for anthocyanidins *FaGT1* resulted in a redirection from anthocyanins to PAs in the flavonoid pathway [42]. Moreover, a previous study has suggested that silencing of *ANR,* the key enzyme for PA biosynthesis in strawberries, lead to a decrease in PA content but to an increase in anthocyanin production at the early fruit-development stage [43]. In contrast, our results show a remarkable increase in *ANR* expression in the PA-treated fruit (Figure 4), and it is reasonable to speculate that exogenous PA treatment induced the *ANR* expression, which therefore up-regulated the endogenous PA content. The results also show that there was an increase after 7 d after 0.4% PA treatment in PA content (Figure 3). We speculate that the reason why the PA content increased at 10 d after treatment is that there was an increase in *LAR* expression at that time. As previously suggested, LAR is one of the key enzymes for PA biosynthesis; the expression pattern of *LAR* genes is consistent with the PAs in strawberries [44]. Overexpression of *LAR* genes could greatly increase the PA content [45,46]. In the meantime, it has been previously reported that overexpression of the *CHS* gene resulted in co-suppression of homologous genes in petunias, and this co-suppression is related to an RNA silencing mechanism [47]; overexpression of apple *ANR* genes inhibited expression of both *CHI* and *DFR* genes, leading to loss of anthocyanin, which might be due to the interactions among enzymes involved in the flavonoid biosynthetic pathway [48,49]. Similarly, in this study, the up-regulation of *ANR* might have induced the down-regulation of *ANS*, *DFR,* and *F3*′*H* at 4 d after PA treatment and correspondingly delayed the accumulation of anthocyanins in strawberry fruit. This result is similar to the result in bananas [26].

Furthermore, fruit softening, another one of the most important ripening traits, is mainly caused by cell wall disassembly and degradation. Pectins represent approximately 60% of the strawberry cell wall [50]; thus, to a great extent, loss of fruit firmness is due to the action of enzymes involved in pectin degradation, such as PG, PL, PME, XYL, EXP, and TFs, including MYB79 and RIF. Among these enzymes, PME provides the hydrolysis substrate for PG and functions synergistically with the PG enzyme to soften fruit [51]; the expression level of *FcEXP1*, *FcEXP2,* and *FcEXP5* was found to be correlated with fruit firmness reduction [52]. Our results show that the firmness loss of control fruit started at 1 d, while under PA treatment, the firmness decrease began at 4 d (Figure 1E), implying the delay of firmness loss with PA treatment. Deeply, we found that the expression of *PL, EXP2, PG2,* and *PME39* was repressed only at 1 d after PAs treatment; the transcript level of *PG1* was inhibited at 1 d and 4 d after PAs treatment; and *PME38* expression was suppressed at 1, 4, and 7 d after PAs treatment (Figure 5). This result indicates that *PG1* and *PME38* might function as important key genes affecting strawberry firmness, as previous studies suggested [53,54]. Furthermore, the RIF encoding a NAC TF and MYB79 was showed to positively regulate strawberry fruit ripening [55,56]. Our result show that the expression of *RIF* was repressed at 1 and 4 d after PA treatment, but increased at 7 d after PA treatment (Figure 5), again indicating that in the PA-treated fruit, fruit ripening was delayed from 4 to 7 d after treatment. The expression of *MYB79* showed no distinct change at 1 and 4 d but significant increase at 7 and 10 d, indicating that *RIF* might play the major role in controlling strawberry fruit ripening; *MYB79* also contributes to strawberry fruit ripening. In addition, lignin is one of the polymers that strengthen plant cell walls and contribute to fruit firmness. The relative expression levels of key structural genes involved in the lignin biosynthesis pathway were examined. It has been suggested that the expression of genes *CAD* and *CCR* strongly influences fruit firmness [57]. Likewise, among the detected lignin biosynthetic genes in this study, *CAD* and *CCR* were found to be significantly inhibited at 4 d after PA treatment; however, most of the other involved genes such as *HCT*, *F5H*, *C3H,* and *CCoAOMT* expressions were not significantly altered at 1 and 4 d but significantly increased at 7 and 10 d after PA treatment (Figure 6). This probably indicates higher lignin levels and explains the decrease in firmness loss in the PA-treated fruit during strawberry ripening.

## 4. Materials and Methods

### 4.1. Plant Materials and Treatment

Strawberry (*Fragaria × ananassa* cv. Benihoppe) fruits were detached from plants at the large green stage from a greenhouse located at Sichuan Agricultural University and transported to the lab immediately. Fruits were selected for uniformity in size and a lack of defects. To keep the water needed for growth, the fruit stalk was enclosed in absorbent cotton and supplied with water each day during the experiment. At least 40 fruits were injected with 1 mL PAs (0.2% and 0.4% *m*/*v*) at the fruit top using a syringe, and another 40 fruits were injected with 1 mL sterile water and used as a control. The injected fruits were placed into an incubator with 25/21 °C (day/night), 16/8 h photoperiod, and 85–90% RH for 10 d. The injection parts of a random 10 fruits were sampled at 1, 4, 7, and 10 d after treatment and then stored at −80 °C for further use. The fruit in the control group (CK) turned to fully red-ripe in 10 days after treatment; to keep the same time period as in the control, we only sampled fruit and detected the physiological changes within 10 days. At least 3 fruits were mixed as one biological replicate, and 3 replicates were detected in total.

### 4.2. Skin Color, Fruit Firmness, TSS, and TA Assays

The fruit skin color was evaluated with a CR-400 chromometer (Konica Minolta, Japan) and represented by L*, a*, and b* values. Two parts of the fruit sides displaying obvious coloring delay were used for the color measurement. Fruit firmness was determined two times on each side of the fruit with a Texture Analyzer TA XT2i (Stable Micro systems, Godalming, Surrey, UK) with a 5 mm diameter cylinder needle. TSS was expressed as percentage and detected using a digital refractometer (PAL-1, Atago, Tokyo, Japan). TA content was presented as citric acid percentage and estimated by titrating the fruit extract against 0.1 M NaOH.

### 4.3. TPC and TFC Evaluations

The TPC and TFC was determined using the Folin–Ciocalteu and aluminum tri-chloride method, respectively, according to previous studies [58,59]. Briefly, around 1 g strawberry samples were extracted in 80% acetone; after standing one hour in room temperature, the mixtures were centrifuged and the supernatant was collected and used for measurement. For TPC detection, 250 μL of the supernatant and 1200 μL of 10% Folin–Ciocalteu reagent (Sigma-Aldrich, St. Louis, MO, USA) were mixed and left to react for 5 min. A solution of 7% sodium carbonate was added and incubated for 30 min at 37 °C to neutralize the mixture. A calibration curve was made by using quercetin as the standard. The absorbance was determined at 415 nm using a UV/vis spectrophotometer. The results were expressed as milligram quercetin equivalent per gram of fresh weight (FW). For TFC determination, 350 μL of the extract was mixed with 150 μL of 5% NaNO_2_, followed by incubation for 6 min. Then, 0.3 mL of 10% aluminum tri-chloride was added, and the mixture was allowed to stand for 5 min. Finally, 1 mol NaOH was added to the mixture and adjusted to 2 mL with distilled water. The absorbance was measured at 650 nm using a UV/vis spectrophotometer. Rutin was used as a standard for constructing the calibration curve. Results were presented as mg of rutin equivalents per g of FW.

### 4.4. Measurement of ABA Content

ABA content was assayed with an Elisa ABA determination Kit (Mlbio, shanghai, China). Strawberry fruit samples of approximately 1 g were extracted with a 9 mL PBS buffer (pH 7.2) and centrifuged at 10,000× *g* for 20 min. The supernatant was used for ABA determination, following the manufacturer’s protocol.

### 4.5. Sugars Detection

Soluble sugars, including sucrose, fructose, and glucose, were evaluated using the modified high-performance liquid chromatography (HPLC) method as previously described [60]. An approximately 0.3 g fruit sample was extracted in 80% ethanol; after standing for 30 min, the mixture was centrifuged for 10 min. The extraction was repeated twice. The supernatant was collected and supplemented to 10 mL by adding sterile water. Then, 2 mL of the supernatant was subjected to a water bath for the evaporation of ethanol. After that, 1 mL sterile water was added and centrifuged for 10 min; the supernatant was filtered with a 0.22 μm membrane and subjected to an HPLC analysis system with a refractive index detector. The separation was achieved using a Platinum Amino column (5 μm, 250 mm × 4.6 mm i.d.; Silgreen, Beijing, China). HPLC analysis was carried out by isocratic elution for 20 min; CAN:H_2_O (80:20, *v*/*v*) was used as the mobile phase, the flow rate was set as 1.2 mL/min, and the injection volume was 10 μL.

### 4.6. PAs and Anthocyanins

An improved DMAC (4-dimethylaminocinnamaldehyde) method was employed to quantify PA content [61]. In brief, approximately 1.5 g fruit samples were extracted with extraction solution consisting of acetone: water: acetic acid = 150:49:1 (*v*/*v*/*v*). After reaction for 1 h, the mixture was centrifuged for 20 min, the supernatant was diluted with 80% ethanol and added to the DMAC solution for quantification at 640 nm using a UV/vis spectrophotometer. Total PA content was expressed as grams of PA per gram of FW.

Anthocyanins were detected by the HPLC method as previously described [62]. In summary, 0.2 g fruit samples were extracted in 2 mL of 1% HCL in methanol for 48 h at 4 °C in darkness. The extraction was repeated once; the clear liquid was collected and filtered using a 0.22 μm membrane. A 10 μL aliquot of the sample was subsequently subjected to HPLC analysis. Compound separation was carried out with an ODS C18 column (5 μm, 250 mm × 4.6 mm i.d., Silgreen, Beijing, China). Methanol and 5% formic acid were used as mobile phases A and B; a linear gradient (95–0%) of A in B was performed for 20 min, followed by 100% B for 5 min. The flow rate was set as 1 mL/min, and chromatograms were recorded at 510 nm. Anthocyanins were quantified by comparing them with external standards. Total anthocyanins were calculated by the sum of Pg3G and Cy3G.

### 4.7. Gene Expression

Total RNA was isolated according to the improved cetyltrimethylammonium bromide (CTAB) method [63]. Frozen fruit samples were finely ground in liquid nitrogen, and extracted with a 3% CTAB extraction solution, containing 100 mM Tris-HCl, 25 mM EDTA, and 2 M NaCl. After a vortex of 3 min, the mixture was placed into a water bath at 65 °C for 30 min and subsequently centrifuged at 4 °C for 10 min. The supernatant was collected into a new clear tube, an equal volume of chloroform was added, it was mixed very well for 5 min, and then centrifuged for 10 min. The extraction with chloroform was repeated three times, and finally the supernatant was precipitated at 4 °C for 1 h using 8 M LiCl. After centrifuging for 10 min and washing with 75% ethanol twice, the RNA pellet was dissolved in DEPC-treated water. The concentration and integrity were measured using a Nano drop and gel electrophoresis. First-strand cDNA was synthesized using a PrimeScript RT reagent Kit (TAKAR, Dalian, China), following the manufacture’s steps.

Quantitative real time PCR (qPCR) reactions were performed in a Bio-Rad 96-well plate system. A total of 10 μL of solution containing 1 μg cDNA, 5 μL SYBR Green PCR Master Mix (TAKARA, Dalian, China), and 10 mM primers was mixed for reaction. The PCR program was set to an initial denaturation at 95 °C for 5 min, denaturation 15 s at 95 °C, annealing 20 s at 58 °C, extension 30 s at 72 °C, and a melting curve from 60 to 95 °C at 0.1 °C/s was recorded. Forty reaction cycles were set. The 26S-18S interspacer RNA was selected as the internal control for normalization. At least two well replicates were performed for each sample, and threeindependent cDNA samples were used as three biological replicates. The primers of flavonoid-related and housekeeping genes were the same as used in a previous study [64]; other primers were designed using NCBI online tool; all primers are listed in Supplementary Table S1.

### 4.8. Statistical Analysis

The relative expression levels of detected genes were calculated by the 2^−ΔΔCT^ method; the 26S-18S interspacer RNA was applied as a reference gene to normalize the expression of the target gene. Experimental data were presented as mean values of three replicates ± standard deviation. Statistics were calculated using the one-way ANOVA method with IBM SPSS Statistics software (v 23.0). The differences between the groups were determined by LSD multiple tests at the significance level of *p* ≤ 0.05.

## 5. Conclusions

The results of this study show that PAs delayed the decrease in strawberry fruit firmness but increased ABA and sucrose content during ripening. Moreover, no obvious regular change patterns of TSS, TPC, and TFC, but a lower TA content, resulted from PA treatment. In addition, PA treatment induced the expression of *ANR*, one of the key genes for PA biosynthesis, and in turn, increased the endogenous PA content. Furthermore, the repression of anthocyanin- and fruit-firmness-related genes by exogenous PAs in the key stage (4 d after treatment, upon which anthocyanins started to accumulate and initiate fruit firmness) could delay the accumulation of anthocyanins and fruit firmness loss during fruit ripening. These findings could be helpful for a better understanding of the biological role of PAs and provide a new strategy to regulate strawberry ripening.

## Figures and Tables

**Table 1:** Effects of PA treatment on fruit TSS, TA, TPC, and TFC. The values are presented as mean ± standard deviation. The lowercase letters indicate the statistical differences at *p* ≤ 0.05 level. TSS: total soluble solids, TA: titratable acidity, TPC: total phenolic content, TFC: total flavonoid content.

	Treatment	0 d	1 d	4 d	7 d	10 d
TSS (%)	CK	7.69 ± 0.60 a	7.29 ± 0.89 ab	6.63 ± 0.74 cd	5.74 ± 0.55 e	5.22 ± 0.34 f
	0.2%	7.69 ± 0.60 a	6.43 ± 0.57 cd	6.79 ± 0.48 bc	6.20 ± 0.41 de	5.20 ± 0.36 f
	0.4%	7.69 ± 0.60 a	6.69 ± 0.50 cd	6.31 ± 0.72 cd	5.7 ± 0.59 de	5.99 ± 0.66 de
TA (%)	CK	0.45 ± 0.04 h	0.67 ± 0.07 fg	0.91 ± 0.10 a	0.82 ± 0.09 bc	0.84 ± 0.04 b
	0.2%	0.45 ± 0.04 h	0.61 ± 0.07 g	0.73 ± 0.03 de	0.71 ± 0.03 def	0.69 ± 0.04 ef
	0.4%	0.45 ± 0.04 h	0.71 ± 0.08 def	0.75 ± 0.06 de	0.70 ± 0.02 ef	0.77 ± 0.03 cd
TPC (g·kg^−1^)	CK	99.06 ± 8.54 bcd	58.60 ± 21.84 e	83.05 ± 31.52 bcde	98.22 ± 22.28 b	127.44 ± 29.10 a
	0.2%	99.06 ± 8.54 bcd	82.41 ± 15.83 bcde	64.90 ± 8.99 de	90.34 ± 15.41 bc	127.96 ± 46.88 a
	0.4%	99.06 ± 8.54 bcd	61.39 ± 8.44 e	68.50 ± 5.02 cde	79.08 ± 12.53 bcde	129.96 ± 44.30 a
TFC (g·kg^−1^)	CK	426.54 ± 16.30 a	322.53 ± 23.47 d	386.24 ± 37.25 ab	391.15 ± 24.32 a	412.54 ± 13.13 a
	0.2%	426.54 ± 16.30 a	344.82 ± 13.15 cd	333.15 ± 24.30 cd	350.92 ± 84.88 bcd	402.01 ± 37.91 a
	0.4%	426.54 ± 16.30 a	329.13 ± 13.17 cd	379.16 ± 15.56 abc	379.56 ± 21.97 abc	396.44 ± 84.10 a

## Data Availability

Not applicable.

## Supplementary Materials

The supporting information can be downloaded at: https://www.mdpi.com/article/10.3390/ijms24043139/s1.
